# A tribute to Stuart A. Kornfeld (1936–2025)

**DOI:** 10.1172/JCI202948

**Published:** 2026-01-02

**Authors:** Richard Steet, Richard D. Cummings

**Affiliations:** 1 Greenwood Genetic Center, Greenwood, South Carolina, USA.; 2 Department of Surgery, Beth Israel Deaconess Medical Center, Harvard Medical School, Boston, Massachusetts, USA.

A giant in the field of biomedical research, Stuart Arthur Kornfeld, MD (Figure 1), passed away in St. Louis on Sunday, August 17, 2025, following complications from Parkinson’s disease. Stuart was the David C. and Betty Farrell Distinguished Professor Emeritus of Medicine at Washington University School of Medicine (WUSM). His leadership roles at WUSM, where he spent his entire clinical research career, included serving as co-director of the Division of Hematology-Oncology from 1976 to 1992 and codirector of the Division of Hematology from 1993 to 2009. Importantly, Stuart directed the Washington University Medical Scientist Training Program (MSTP) from 1991to 1997. He was also the founder in 2000 of the first physician-scientist training program (PSTP) in the United States. His commitment to research and the training of physician-scientists was monumental and historic. Stuart was one of the founders of the field of glycobiology, as well as being the one of the first to identify clinical causes of diseases related to protein glycosylation and modification. His studies on glycoprotein biosynthesis, the pathways of glycosylating asparagine residues, and the functions of the oligosaccharides (glycans) and his discovery of their roles in regulating trafficking of lysosomal hydrolases to the lysosome — as well as the formation of that organelle — were groundbreaking (1, 2). He was the quintessential physician-scientist, and one could imagine the term having been created in his honor.

Stuart was born in St. Louis, Missouri, on October 4, 1936. Stuart’s father, Max, was a dentist and faculty member at Washington University in St. Louis, where he taught and conducted research. Stuart’s mother, Ruth, was his father’s laboratory assistant. Stuart had an intense interest in sports, played tennis and golf throughout his life, and loved baseball. He was an avid, lifelong fan of the St. Louis Cardinals and could easily be drawn into deep conversations about batting averages. But while Stuart was interested in sports and architecture, and imagined following in the footsteps of Frank Lloyd Wright, his family encouraged him to go into medicine (3). He enrolled at Dartmouth, where he graduated in 1958 with a degree in zoology. During that time, he was impressed by textbook articles on metabolism from the incredible duo of Gerty and Carl Cori, who in 1931 had become faculty members at WUSM. The Coris were among the few married couples to be awarded the Nobel Prize (1947) for their work on glucose metabolism. This encouraged Stuart to enroll at WUSM, from which he received his MD in 1962. While a medical student, he found time to work with Luis Glaser, who had worked with the Coris and whose lab was next door to Gerty Cori’s. Among the highlights of his student days at WUSM was meeting a young PhD student named Rosalind Hauk. They married in 1959, and a new powerhouse research duo was created. They worked together for 48 years until Rosalind’s passing in 2007. Following his clinical training at WUSM, and based on suggestions from Carl V. Moore, Chair of Medicine, and Carl Cori, both Stuart and Rosalind moved to Washington, DC, and the NIH. Here Stuart, and later Rosalind, worked with Victor Ginsburg, one of the founding biochemists in the field of glycobiology. During this time, Stuart developed his interest in the ABO blood group antigens and antibodies and lectins that recognized the antigenic sugars. After successful studies at the NIH, Stuart and Rosalind moved back to WUSM, where they set up a joint lab. At the time of Stuart’s hiring, Carl Moore also hired another young clinical researcher named Philip Majerus. Stuart and Phil became lifelong friends and eventually coleaders of hematology at WUSM.

At WUSM, Stuart initiated his research on immunoglobulin glycosylation and the nature of glycan recognition by lectins, as well as how such interactions with glycoproteins and blood cells are specific in terms of carbohydrate recognition (4, 5). These studies focused on glycan structures linked to Asn (Asn-linked oligosaccharides, or N-glycans) and to Ser/Thr (O-linked oligosaccharides, or O-glycans) and led to remarkable insights about specificities of the glycosylation machinery. Stuart took the unusual approach of exploiting the killing activity of the extremely toxic plant lectin ricin, which led to identification of a ricin-resistant clone of Chinese hamster ovary cells (CHO clone 15B). These cells produced glycans lacking complex N-glycans and associated galactose or sialic acid residues, and hence were not recognized by ricin, which requires these residues for binding. 15B cells generated what is now termed oligomannose type N-glycans, because they lack the key enzyme N-acetylglucosaminyltransferase I, which initiates conversion of oligomannose to hybrid-type N-glycans on the pathway to complex-type chains. This mutant cell line and many others also developed by the Kornfeld laboratory inspired studies on biosynthetic pathways at the genetic level, including those of James Rothman in vesicular transport, and eventually definition of the lipid-linked oligosaccharide precursors of oligomannose in N-glycans. These studies were aided by creative use of radiolabeled sugar precursors, e.g., 2-^3^H-mannose; isolation and characterization of newly synthesized, radiolabeled glycans were keys to these and many other insights from Kornfeld’s group. Kornfeld’s seminal publications describing the biosynthetic maturation of glycans and their complex pathways represented a tour de force in the late 1970s. His studies laid the foundation for our current understanding of glycoprotein biosynthesis involving the endoplasmic reticulum, Golgi apparatus, and overall secretory pathway. A key review article on this subject by Stuart and Rosalind Kornfeld in 1985 has been cited over 4,500 times and counting (6).

In these studies, Kornfeld’s group identified that the N-glycans of the enzyme β-glucuronidase contain phosphorylated mannose residues occurring in two versions: GlcNAc1-P-Man-R and P-Man-R, a phosphodiester and phosphomonoester, respectively. The former was created by the enzyme his lab discovered and is abbreviated as N-acetylglucosaminylphosphotransferase; and for the latter, the GlcNAc was removed by the “uncovering enzyme,” an α–N-acetylglucosaminidase. Fibroblasts from children with mucolipidosis II (I-cell) disease were shown to lack the N-acetylglucosaminylphosphotransferase, a breakthrough in glycobiology and clinical medicine (7). Following this discovery of the mannose-6-phosphate tag (8), the efforts of Stuart’s laboratory shifted toward receptors that bind this tag and direct acid hydrolases to lysosomes. His laboratory was instrumental during this phase of discovery, reporting cloning and characterization of both cation-independent and cation-dependent M6P receptors in the late 1980s. Stuart’s group also defined the M6P-binding sites in these receptors, as well as the signals in the cytoplasmic tails responsible for rapid internalization. In a manner that characterized Stuart’s entire scientific career, solving one problem invariably led him to pursue the next logical question — in this case, how exactly the receptors were packaged into clathrin-coated vesicles. Over the next two decades, Stuart’s lab made significant advances in our understanding of adaptor proteins and GGAs, the ARF-dependent clathrin adaptors that mediate trafficking and recycling of M6P receptors.

While work on the M6P receptors proceeded, his lab also focused on trying to understand how the phosphotransferase enzyme specifically recognizes lysosomal enzymes. Much of this work involved site-directed mutagenesis of a model hydrolase, cathepsin D, wherein he explored the role of key lysine residues in establishing this specificity. This area of research would continue in Stuart’s lab until recently, when he and others solved the cryo-EM structure of the enzyme and defined the regulatory mechanism for this remarkable specificity. This investigation also culminated in the discovery of a truncated form of the phosphotransferase enzyme (termed S1S3) that exhibited increased ability to phosphorylate lysosomal enzymes. Such a discovery opened the door for use of the S1S3 enzyme for production of therapeutic enzymes with improved uptake into cells, among other biotechnological uses.

Stuart’s research would eventually lead him back to medicine and investigation of disease mechanisms associated with impaired M6P-dependent trafficking of lysosomal enzymes. With the advent of genome editing and development of new animal models for mucolipidosis II, Stuart began a systematic characterization of the phenotypes associated with loss of *GNPTAB* and *GNPTG*. These studies uncovered new structural motifs in the enzyme, revealed how different mutations found in *GNPTAB* cause dysfunction of the phosphotransferase enzyme, and characterized new proteins such as TMEM251 involved in processing the enzyme into its mature form.

The universal attributes of Stuart’s research that every trainee who passed through his lab would affirm is the strength of his scientific logic and methodical approach, and his ability to never lose sight of the patient and the medical implications of his research. Stuart held one of the longest-standing NIH grants for his entire 50-plus-year career and was the recipient of numerous awards, including the Passano Award and the Kober Medal (9). He was an elected member of the National Academy of Medicine, the National Academy of Sciences, the American Society for Clinical Investigation, the Association of American Physicians, and the American Academy of Arts and Sciences. He is also known for having trained countless independent scientists who have gone on to highly successful careers and leadership roles. All trainees fondly recall the time they spent in Stuart’s lab and the insight they gained from his mentorship. Stuart’s family life was just as important to him as his professional life. Stuart and Rosalind had 3 children — youngest daughter, Carolyn Kornfeld Lesorogol, PhD, a professor in the Brown School at Washington University, who predeceased him; daughter Katherine Kornfeld, senior director of foundation relations at Washington University; and son Kerry Kornfeld, MD, PhD, a professor in developmental biology at WUSM. Stuart is also survived by six grandchildren; two great-grandchildren; and his beloved friend of many years, Elizabeth Loeb. Stuart will forever be remembered for his integrity, kind and generous manner, gentle approach to friends and colleagues, and uncanny natural intuitiveness in clinical and basic science research.

## Figures and Tables

**Figure 1:**
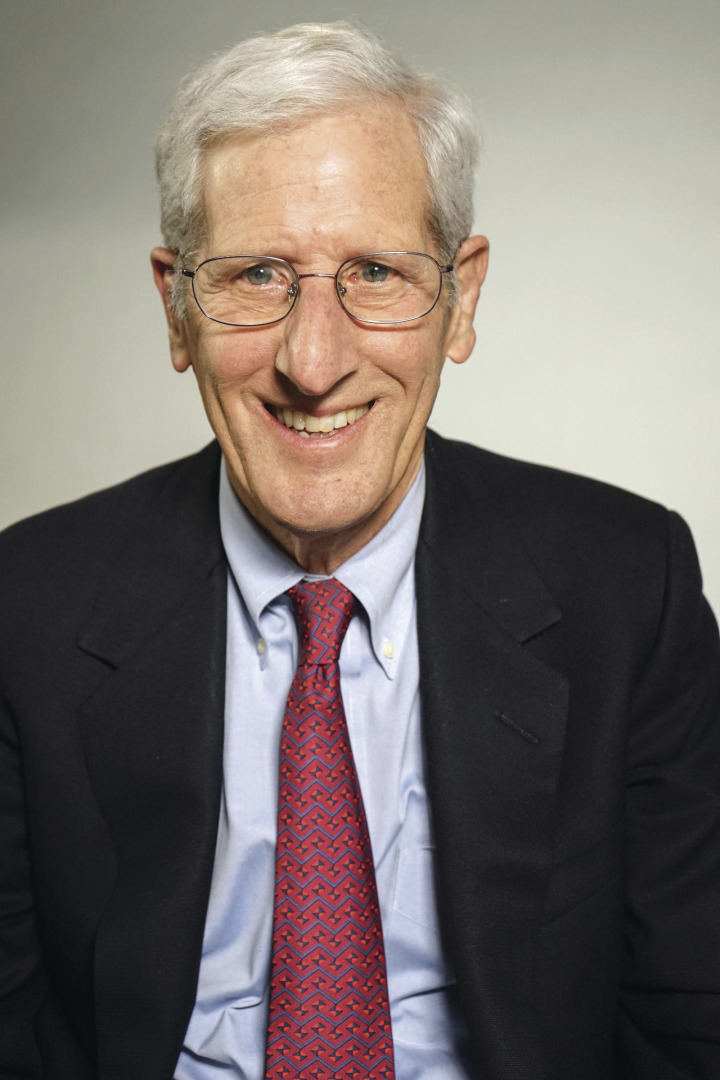
Stuart A. Kornfeld, MD. Image credit: Karen Guth.

## Version 1 01/02/2026

Electronic publication
